# Bone microstructure of the basal anomodont *Suminia getmanovi* supports its arboreal lifestyle

**DOI:** 10.1038/s41598-025-92727-w

**Published:** 2025-03-25

**Authors:** Saskia Nieke, Jörg Fröbisch, Aurore Canoville

**Affiliations:** 1 Museum für Naturkunde Leibniz-Institut für Evolutions- und Biodiversitätsforschung, Berlin, Germany; 2 Humboldt-Universität zu Berlin, Berlin, Germany; 3 Friedenstein Stiftung Gotha, Gotha, Germany

**Keywords:** Bone microanatomy, Ontogenetic stage, Growth strategy, Climbing, Paleoecological inferences, Evolutionary ecology, Palaeoecology

## Abstract

The paleohistology of Permo-Triassic anomodonts has been extensively studied and, independent of phylogeny, body size and lifestyle, reflects a pattern of rapid growth indicated by a woven-parallel complex. Moreover, anomodonts uniformly show a relative bone cortical thickness (RBT) exceeding 30% and a medullary cavity generally filled by trabeculae. Here, we investigate the paleohistology of the basal anomodont *Suminia getmanovi* from the Permian of Russia, which has been hypothesized as one of the earliest arboreal tetrapods. Osteohistology and skeletal proportions reveal that our sample comprises at least two late juvenile to early subadult individuals, exhibiting well-vascularized and mostly uninterrupted woven-parallel complex or parallel-fibered tissues, suggesting relatively high growth rates, consistent with other anomodonts. However, all elements of *Suminia* present an open medullary cavity virtually free of bony trabeculae and a RBT lower than 18%. The microanatomy of *Suminia* thus differs from all other anomodonts studied so far, including its closest relative *Galeops*, as well as more basal synapsids that also tend to show higher RBT values and/or a medullary territory obstructed by trabeculae. Compared to extant climbers, which possess thinner bone walls and lower compactness than their terrestrial and aquatic relatives, the bone architecture of *Suminia* further supports its arboreal lifestyle.

## Introduction

**Fig. 1:**
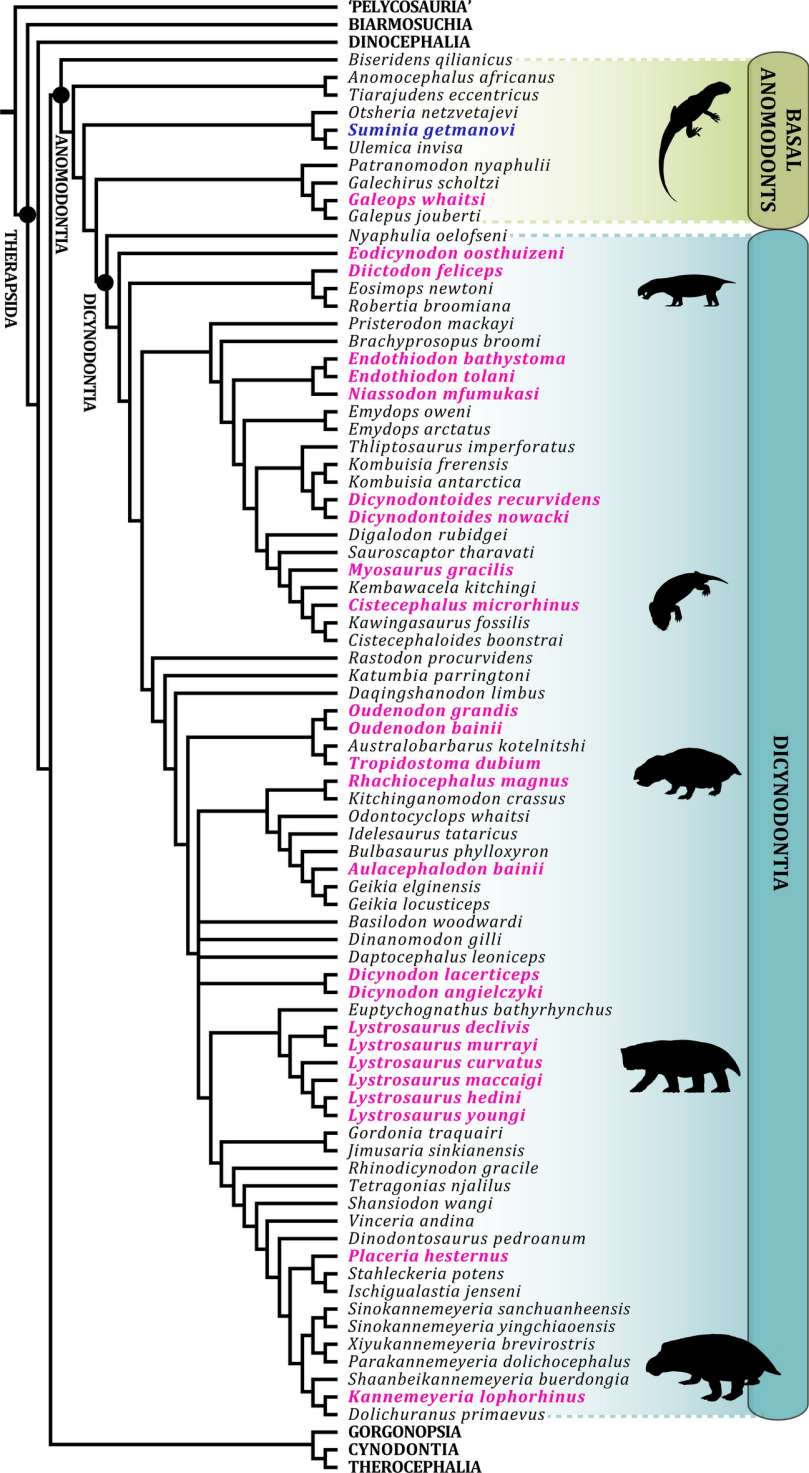
Simplified phylogeny of Synapsida with an emphasis on Anomodontia diversity (modified from^20^: fig. 25). *Suminia getmanovi*, the taxon of interest in this study, is highlighted in blue. Anomodont species whose long bone paleohistology has been previously investigated are highlighted in magenta.

Anomodontia not only represented the major primary consumers of Permo-Triassic continental ecosystems^ 1^, but also constituted the most widespread and disparate group of non-mammalian synapsids ^2–4^. Their fossil record is, however, quite heterogeneous. The derived and very speciose Dicynodontia, a clade generally characterized by the presence of two upper caniniform teeth, often referred to as tusks ^4^, and otherwise mostly edentulous jaws that were covered by a keratinous beak, are known from often abundant and well-preserved remains ^5^ collected across all continents ^6,7^. As a result, their paleobiology has been fairly well studied based on morphology (e.g.^8–11^), taphonomy^ 12,13^, but also long bone and tooth paleohistology (see^14,15^ and references therein; Fig. 1). Despite their unparalleled eco-morphological diversity, ranging from mole-sized fossorial forms (e.g. cistecephalids^9,16^) up to elephant-sized graviportal grazers (e.g. *Lisowicia*^17^), they notably show consistent growth patterns (e.g.^18^). Their well-vascularized cortices primarily made of woven bone tissue, combined with the absence of growth marks (i.e. zones, annuli, and lines of arrested growth [LAG]) early in ontogeny (up to 50–70% of adult size, depending on the species), indicate sustained and relatively fast growth up to a sub-adult stage. Regardless of specific body-size, long bone microanatomy is also homogeneous across dicynodonts. Most sampled species show thick compact cortices in their stylopodial and zeugopodial elements, with a relative bone wall thickness (RBT;^19^) superior to 30% in subadult (individuals > 40% of adult size according to ^18^) and adult individuals. This is usually associated with a medullary territory obstructed by a network of trabeculae, resulting in an overall compact bone architecture^ 18^.

To the contrary, basal non-dicynodont anomodonts have received very little research attention because of sparse and often incomplete remains. Most species (about 10 genera recognized to date;^20^) are only known from cranial and/or partial postcranial material of one or just a few specimens^20,21^. Although they do not form a monophyletic group, they have been described as slender and seemingly more agile than their dicynodont relatives, with a plesiomorphic long tail and complete tooth rows in both upper and lower jaws^4^. Their paleobiology and paleohistology remain however poorly investigated, with the exception of *Galeops whaitsi* (Fig. 1), retrieved as one of the closest relatives to Dicynodontia by several studies^20,22^. Botha-Brink and Angielczyk^18^ sampled the humerus, femur, tibia and fibula of a fully-grown individual (SAM-PK-12261) and found that its osteohistology is comparable to dicynodonts’. *Galeops* shows cortices formed primarily of a woven-parallel complex, although rather poorly vascularized, interrupted by growth marks. RBT values are above 34% in all sampled elements.

**Fig. 2:**
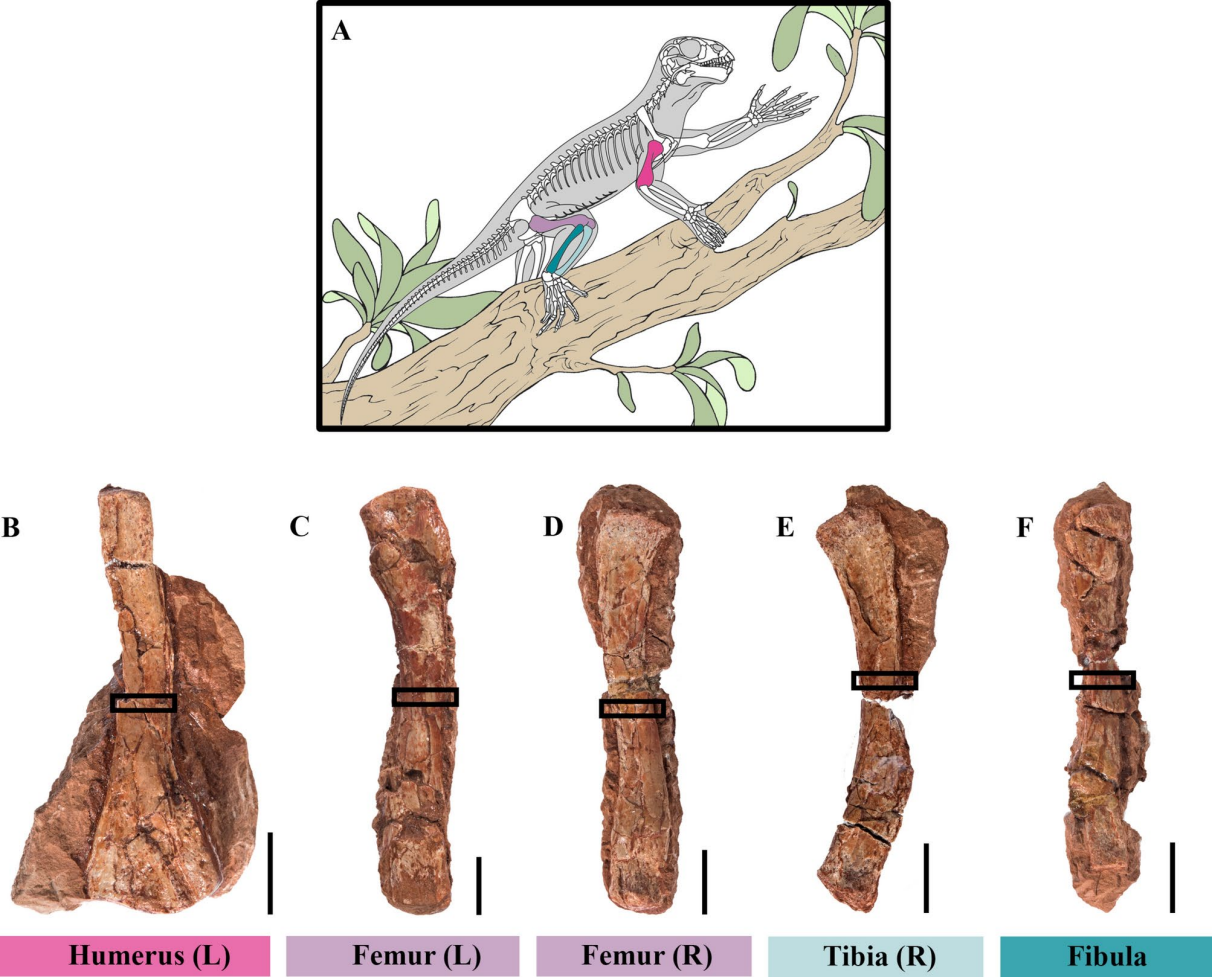
Reconstruction of *Suminia getmanovi* as arboreal (**A**; modified from^21^: fig. 16) and limb bone sample investigated (specimen KPM 20/99; **B–F**). The mid-diaphyseal areas sampled are marked by black rectangles. Scale bars equal 1 cm.

Among basal anomodonts, *Suminia getmanovi*^23^ is a particularly relevant taxon to expand on the osteohistological sampling of this group. This species is by far the best-represented basal form with over 70 discovered specimens^24,25^, many of which are sub-complete and articulated individuals^21,23–26^. Moreover, its skeletal anatomy is highly derived. *Suminia* has been inferred as the oldest known herbivore with efficient oral processing of high-fiber plant material, based on its occluding dentition, leaf-shaped teeth with serrated edges and wear facets^26,27^. It is also hypothesized to be one of the oldest arboreal, and therefore climbing tetrapods (^21,24^; Fig. 2A). This lifestyle inference relies on a suite of morphological characteristics, including elongated limbs and phalanges, long and laterally compressed unguals coupled with a divergent first digit on both manus and pes suggesting grasping abilities, and a potentially prehensile tail^21,24^.

Here, we investigate for the first time the bone microstructure of *Suminia getmanovi*, based on a sample of five isolated limb bones all catalogued under specimen number KPM 20/99 (Fig. 2B–F). The goals of our study are manifold: (1) We aim to better characterize the composition of our sample by testing the hypothesis that all isolated bones belonged to a single individual. To do so, we will compare the bone histology and inferred ontogenetic stages of the different limb elements^28,29^. In addition, the length ratios between the sampled bones will be compared to published ratios calculated from fully articulated *Suminia* specimens (e.g.^21^: appendix 1); (2) Second, limb bone histology of *Suminia* will help us decipher the growth strategy of this taxon that will be subsequently compared to the relatively high growth rates documented in *Galeops* and the more derived dicynodonts^18^; (3) The long bone microanatomy (i.e., overall inner bone architecture) of *Suminia* will also be evaluated, using different quantitative parameters commonly used in the literature^18,30^, in order to further test the hypothesis of a clinging arboreal lifestyle in this basal anomodont. An extensive body of work explored the relationship between lifestyle habits and bone microanatomy (see^31^ and references therein) and showed that semi-aquatic, terrestrial, and fossorial tetrapods exhibit thicker cortices and overall more compact bone architectures than their flying counterparts. Flying taxa (flying birds, bats, pterosaurs and other gliding reptiles) that have to lift their body weight against gravity, possess lightly built limb bones with a relatively enlarged medullary cavity, mostly free of bone trabeculae, and very thin bone walls^32–35^. Although the effect of an arboreal lifestyle on limb bone microanatomy has not been extensively investigated, studies have shown that, for example, scansorial mustelid species exhibit different bone cross-sectional traits than their fossorial, natatorial, terrestrial, or even generalist relatives, including significantly lower cortical thickness and overall compactness values^36,37^. An overall lower body mass is indeed advantageous to counteract gravitational and inertial forces during vertical ascent^38^. If *Suminia* were truly arboreal, we would thus predict thinner cortices (i.e. lower RBT values) and wider medullary cavities in its limb bones, as compared to other anomodonts that have mostly been inferred terrestrial or fossorial^39,40^. To support an arboreal lifestyle, the overall bone microanatomy of *Suminia* should also differ from the expected plesiomorphic condition for anomodonts; (4) Additionally, we will apply different lifestyle^41–43^, and body mass^44^ inference models available in the literature to our sample in order to gather further information related to the paleobiology of *Suminia*. Ultimately, this new bone microstructural study of *Suminia* will contribute to better documenting the osteohistological diversity of basal anomodonts and therapsids more generally.

## Results

### Limb bone measurements and proportions

With a length of 53.5 mm, the left humerus of KPM 20/99 falls into the smaller range of the documented size for *Suminia getmanovi* humerii (Fig. S1A). Its length nonetheless corresponds to 85.3% of one of the largest individuals known for *Suminia* (PIN 2212/116, Specimen 5;^21^). The left KPM 20/99 femur falls within the larger range of known femora, with a length of 75.8 mm, representing 93.7% of the largest femur (PIN 2212/116, Specimen 5). The right KPM 20/99 femur, on the other hand, is among the smallest femora documented for *Suminia*, with a length of 64.3 mm, reaching 79.5% of PIN 2212/116, Specimen 5. The right tibia of KPM 20/99 has an approximate length of 59.4 mm, which is about 96.6% of the tibia of PIN 22122/116, Specimen 5. Finally, the fibula is the smallest documented so far, reaching approximately 80% of the fibula of PIN 2212/116, Specimen 5.

In order to decipher whether the sampled skeletal elements belonged to one or multiple individuals, we also compared the ratios between these bones to the ratios calculated from articulated individuals of *Suminia* (i.e., specimens PIN 2212/116, PIN 2212/102, ROM 80979; Fig. S1). In this species, the femur is longer than the humerus. The ratio between the stylopod lengths varies slightly from one individual to another, but *Lg*_*femur*_/*Lg*_*humerus*_ is on average around 1.29. The ratios between the KPM 20/99 humerus and both femora do not fall within the range of articulated specimens, but they are relatively similar (Fig. S1A). Similarly, femora are usually longer than the associated tibia with a ratio averaging 1.28 (Fig. S1B). In KPM 20/99, the length ratio between the large femur and the tibia is 1.28 and indicates that these skeletal elements might have belonged to the same individual. However, the length ratio between the small femur and the tibia falls well outside the documented range for *Suminia* with a value of 1.08, indicating that these elements most likely did not belong to the same individual. Similarly, the length ratio between the large femur and the fibula does not fall within the range documented in articulated specimens, suggesting that these elements might not come from the same individual. However, the ratio between the small femur and the fibula falls well within the range documented in other specimens and suggests that these elements might have belonged to one individual. Finally, the ratio between the KPM 20/99 tibia and the fibula is significantly different from the range known from other articulated specimens, confirming that these elements did not belong to the same individual.

### Limb bone histology

**Fig. 3:**
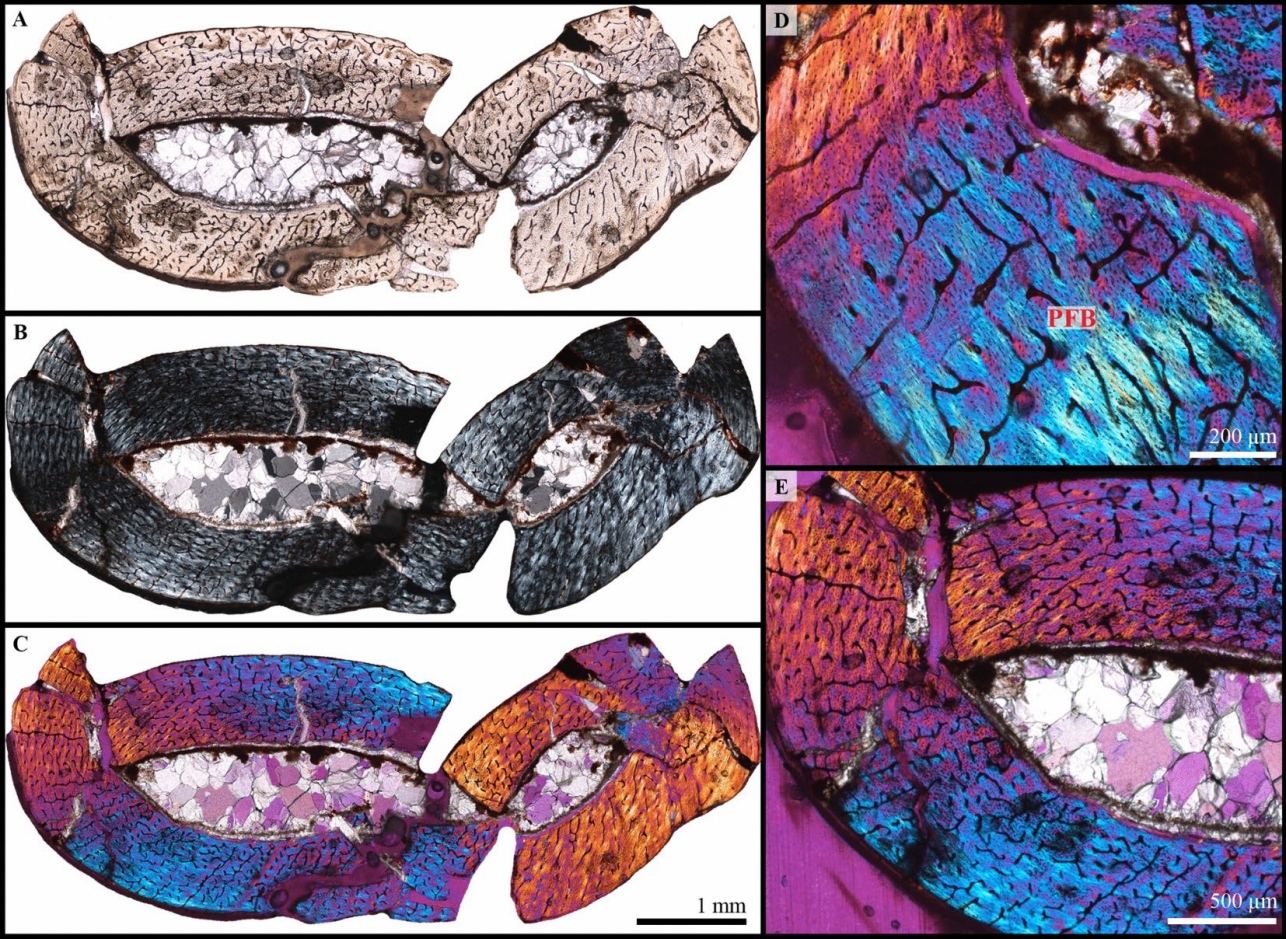
Osteohistology of KPM 20/99 left humerus. Complete mid-diaphyseal cross-section in direct light (**A**), polarized light (**B**) and polarized light with a lambda compensator (**C**). (**D**, **E**) close ups showing the periosteal cortex primarily composed of a well-vascularized parallel-fibered bone tissue (PFB). Vascular canals (simple vascular canals and primary osteons) have a longitudinal, radial or reticular orientation. The endosteal margin is resorptive and remodeling is limited.

Humerus (left)—All mid-diaphyseal cross-sections obtained for this bone are fairly complete, but crushed (Fig. 3A–C). However, the bone histology is relatively well preserved and homogeneous in all the sections. The medullary cavity is void of sediment matrix, but is instead filled with recrystallizations (Fig. 3A–C), and seems to have been almost free of bony trabeculae during life (with the exception of a few isolated bone struts). The compact cortex is mostly formed of an uninterrupted (complete absence of growth marks, i.e. zones, annuli, and LAGs) and highly vascularized parallel-fibered bone tissue with some patches of woven-parallel complex in the deep cortex (Fig. 3D, E). Vascularization is composed of primary osteons and simple vascular canals, with a longitudinal and reticular organization and a few noticeable radial canals running throughout the cortex (Fig. 3D, E). Although some vascular canals still pierce the periosteal surface, a very slight decrease in the density of vascularization is visible towards the bone periphery. This is associated with a subtle change in osteocyte lacuna density and shape. Indeed, osteocyte lacunae tend to be plump and numerous in the deep and mid-cortex. Towards the periphery, their density decreases and they become more flattened. The endosteal margin is still resorptive and no lamellar endosteal bone tissue is observable, showing that the expansion of the medullary cavity had not yet ceased (Fig. 3D). Finally, secondary remodeling is very limited in the cortex and restricted to a few isolated and small secondary osteons.

**Fig. 4:**
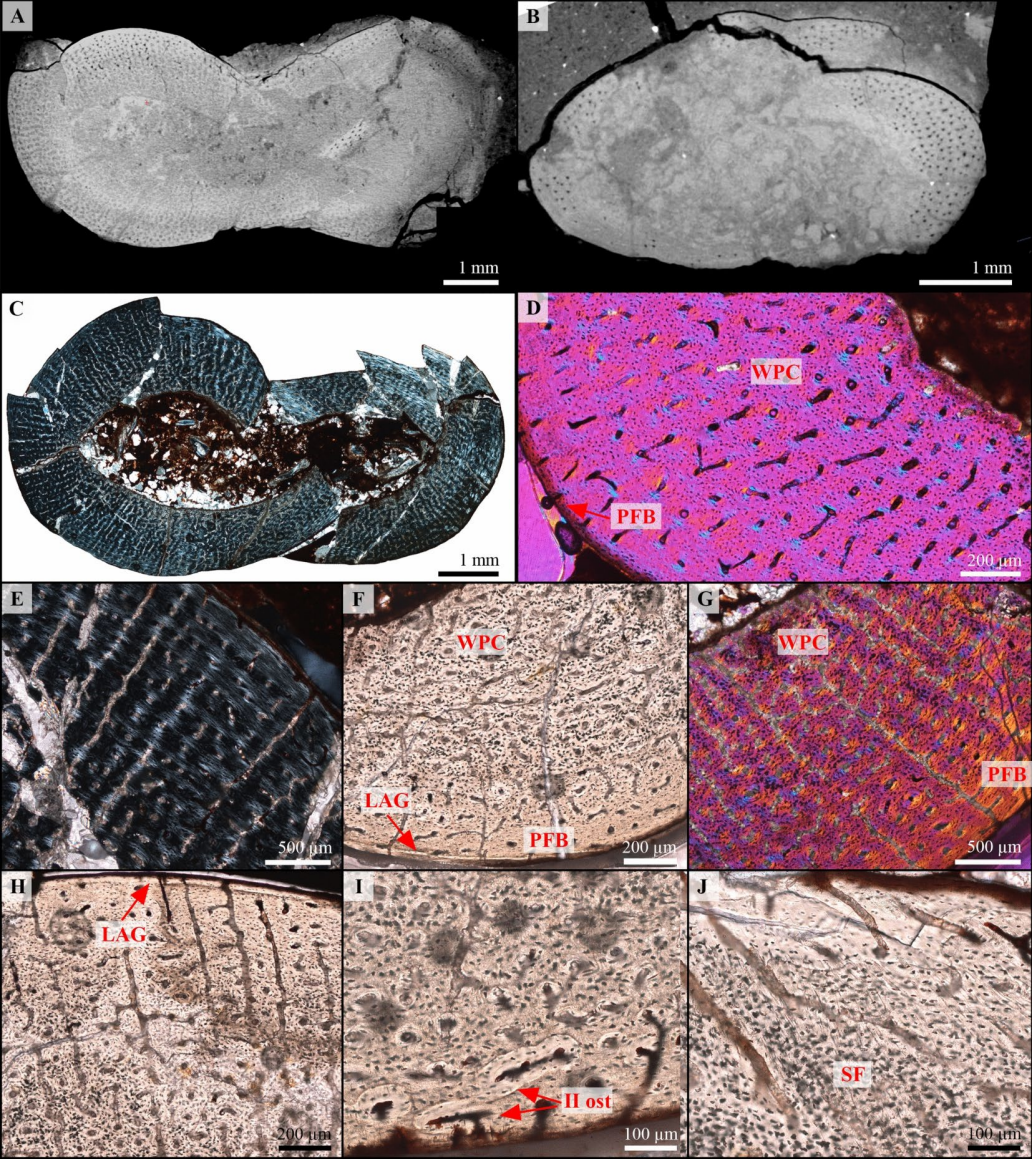
Osteohistology of both KPM 20/99 femora (**A**, **C**, **E–J**: larger left femur; **B**,** D**: smaller right femur). (**A**) Mid-diaphyseal virtual section of the larger left femur. Note the organization of the numerous longitudinal vascular canals in radial rows. (**B**) Mid-diaphyseal virtual section of the smaller right femur. The vascular canals have a preferential longitudinal orientation. (**C**) Complete mid-diaphyseal cross-section of the larger left femur (in polarized light). Vascular canals are organized in radial rows. (**D**) Close up of the smaller right femur cortex mostly comprised of a woven-parallel complex (WPC) with longitudinally oriented primary osteons and some radial anastomoses (in polarized light with lambda compensator). Note the presence of a thin region of more parallel-fibered bone tissue (PFB) with flattened and more organized osteocyte lacunae at the sub-periosteal surface. The endosteal margin is resorptive and remodeling is absent. (**E**) Close up of the cortex of the larger left femur (in polarized light). The cortex consists of a well-vascularized woven-parallel cortex (WPC; deep cortex) and parallel-fibered bone tissue (PFB; outer cortex) with longitudinally oriented primary osteons organized in radial rows, and some radial canals. (**F**) Close up of the cortex of the larger left femur highlighting the decrease in vascularization and osteocyte lacuna density towards the bone periphery. A faint line of arrested growth (LAG) is also visible at the periosteal surface. (**G**) Similar to D (in polarized light with lambda compensator). Note the presence of some radial canals and radial anastomoses running through most of the cortex, as well as some secondary osteons in the external cortex. (**H**) Close up of the cortex of the larger left femur. Note the presence of radial canals and anastomoses, as well as a LAG in the outermost cortex. (**I**) Close up of the outer cortex of the larger left femur showing secondary osteons (II ost). (**J**) Close up of the outer cortex of the larger left femur showing Sharpey’s fibers (SF) associated with numerous and plump osteocyte lacunae.

Larger femur (left)—This femur has also been diagenetically altered. The mid-diaphyseal cross-sections obtained are fairly complete, although crushed (Fig. 4A, C). As in the humerus, the cortex is compact and the transition with the medullary cavity is abrupt and well defined. The medullary cavity is filled with sediment, recrystallizations and isolated trabecular fragments (Fig. 4C). This shows that the medullary cavity was wide open, with only a few scattered trabeculae. The inner 75% of the cortex consists of a highly vascularized woven-parallel complex (Fig. 4E–G). The primary osteons have a preferential longitudinal orientation, but organized in radial rows, in some parts of the section or have a more reticular organization in others (Fig. 4E, F). Numerous radial canals and anastomoses also run from the deep to the outer cortex (Fig. 4E, G, H). There is a very clear decrease in bone depositional rate towards the periphery, with a decrease in vascularization in the outer quarter and gradual increase in parallel fibered bone (Fig. 4F, G). This is accompanied by a decrease in the density of osteocyte lacunae that are plump, numerous and disorganized in the deep and middle cortex, but smaller, fusiform and better organized towards the periphery (Fig. 4F). Sharpey’s fibers appear in localized patches, oblique to the cortical surface, in areas where the cortex is the thinnest (Fig. 4J). A single LAG, i.e. a clear discontinuity in the bone tissue marking a temporary cessation of growth^28^, is visible in some well-preserved portions of the outermost cortex, just under the periosteal surface (Fig. 4F, H). The endosteal margin is still resorptive and no endosteal lamellar bone has formed (Fig. 4C, F, G). Secondary remodeling is limited to a few secondary osteons (Fig. 4G, I).

Smaller femur (right)—The bone was highly damaged during fossilization; all obtained mid-diaphyseal cross-sections are incomplete (Fig. 4B). The medullary cavity is infilled with sediment, recrystallizations, and numerous bone shards originating from damaged parts of the inner cortex. As for the other femur and the humerus, the bone wall is compact and its interface with the medullary cavity is well defined (Fig. 4B, D). The cortex is mostly formed of a well-vascularized woven-parallel complex with some patches of more parallel-fibered tissue (Fig. 4D). Primary osteons have a preferential longitudinal orientation, but they are arranged in somewhat radial rows with some radial anastomoses (Fig. 4D). In the thin outermost periosteal region, the tissue is slightly more organized with osteocyte lacunae becoming parallel to each other and fusiform (Fig. 4D). However, vascular canals still pierce through the bone surface. Sharpey’s fibers are visible in some localized areas of the cortex. Finally, secondary remodeling is absent and the endosteal margin still resorptive. No growth marks are visible.

**Fig. 5:**
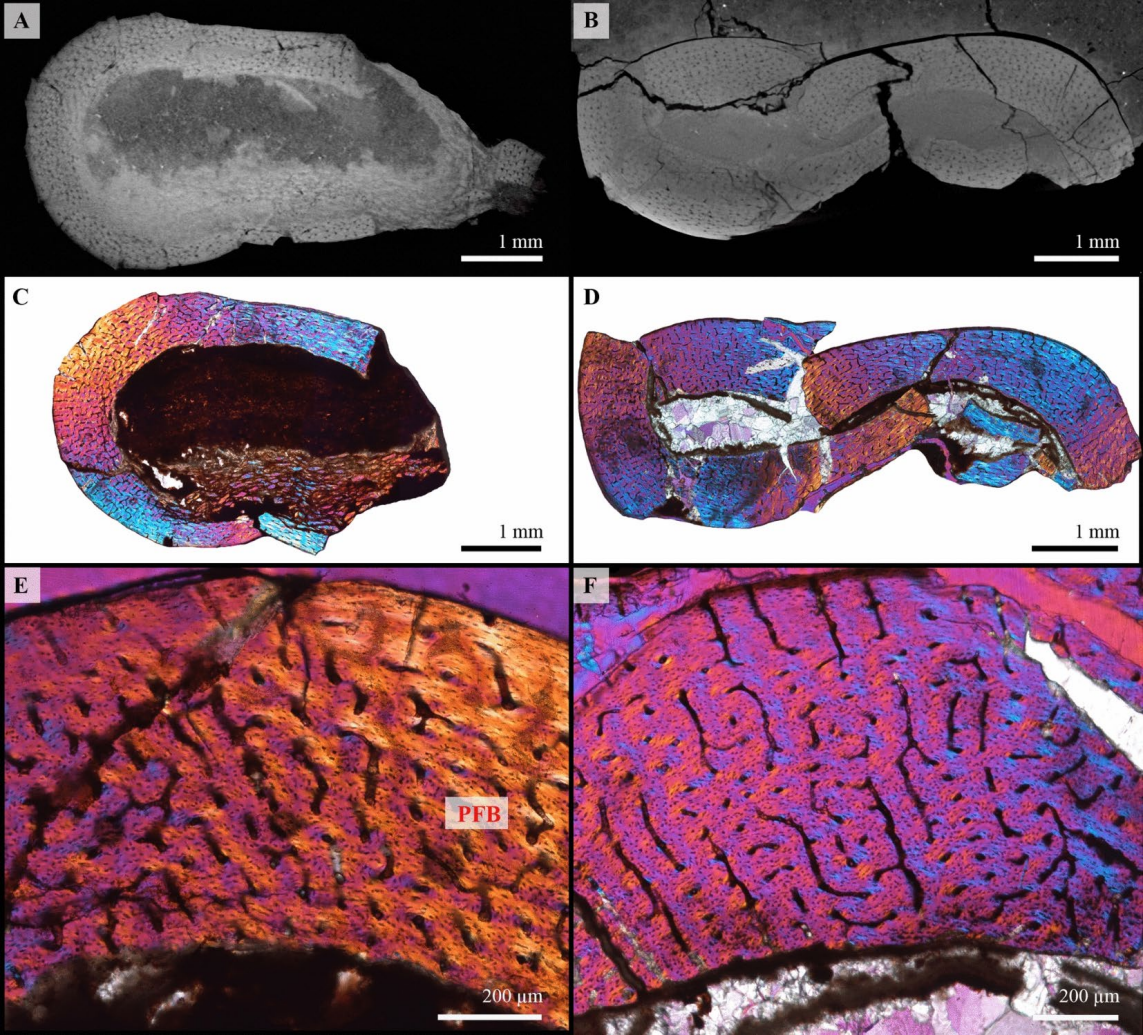
Osteohistology of KPM 20/99 right tibia (**A**, **C**, **E**) and fibula (**B**, **D**, **F**). (**A**) Virtual transverse section of right tibia. The cortex is well-vascularized. (**B**) Virtual transverse section of fibula. The cortex is well vascularized and the longitudinal canals are organized in radial rows. (**C**) Partial cross-section of right tibia (in polarized light with lambda compensator). The preserved cortex is mostly parallel-fibered with a reticular vascularization. (**D**) Cross-section of fibula (in polarized light with lambda compensator). Depending on the area of the section, the periosteal cortex is formed of a well-vascularized parallel-fibered bone matrix or a woven parallel-complex. Vascular canals have a preferential radial or longitudinal orientation. (**E**) Close up of cortex in the right tibia (in polarized light with lambda compensator) mostly composed of parallel-fibered bone tissue (PFB). Note the slight decrease in vascularization and osteocyte lacuna density towards the bone periphery. The endosteal margin is resorptive and secondary remodeling is limited. (**F**) Close up of the cortex in the fibula (in polarized light with lambda compensator). Note the slight decrease in vascularization and osteocyte lacuna density towards the bone periphery. The endosteal margin is resorptive and secondary remodeling is limited.

Tibia (right)—Again, this element was damaged and crushed during fossilization and thus, all mid-diaphyseal cross-sections are incomplete (Fig. 5A, C). The medullary cavity is infilled with sediment, recrystallizations, and numerous bone fragments resulting from breakage of the inner cortex (Fig. 5A, C). In areas that were minimally altered by diagenesis, the bone wall is compact and its transition with the medullary cavity well defined and abrupt (Fig. 5E). No trabeculae are visible in the wide-open medullary cavity. The cortex is comprised of a mix of woven and parallel-fibered bone matrix which is well vascularized (Fig. 5C, E). Primary osteons and simple vascular canals have a preferential longitudinal or reticular orientation, depending on the area of the section (Fig. 5C, E). The thin sub-periosteal bone area tends to be more organized with flattened osteocyte lacunae that run parallel to each other. Some long Sharpey’s fibers are present in areas where the cortex is the thinnest. The endosteal margin is still resorptive and secondary remodeling is restricted to some scattered secondary osteons.

Fibula—Despite being crushed, this bone cross-section is fairly complete (Fig. 5B, D). The medullary cavity is mostly obstructed by recrystallizations and there are no visible remnants of trabeculae, suggesting that the cavity was wide open when the animal was alive (Fig. 5D). As in the other limb elements, the cortex is compact and the endosteal margin well defined, although still resorptive (Fig. 5F). Depending on the area of the section, the bone is comprised of a well-vascularized parallel-fibered bone and patches of a woven-parallel complex. The orientation of primary osteons varies throughout the section and may be longitudinal or reticular. Radial anastomoses are also prevalent throughout the section (Fig. 5F). As in other skeletal elements, there is a subtle and progressive decrease in the density and volume of osteocyte lacunae from the deep to the outer cortex. In the outermost cortical region, osteocyte lacunae tend to be flattened and parallel to each other, suggesting a decrease in growth (Fig. 5F). Vascular canals still pierce the periosteal surface. Finally, growth marks or secondary remodeling were not observed in this bone.

### Mid-diaphyseal microanatomy

**Fig. 6:**
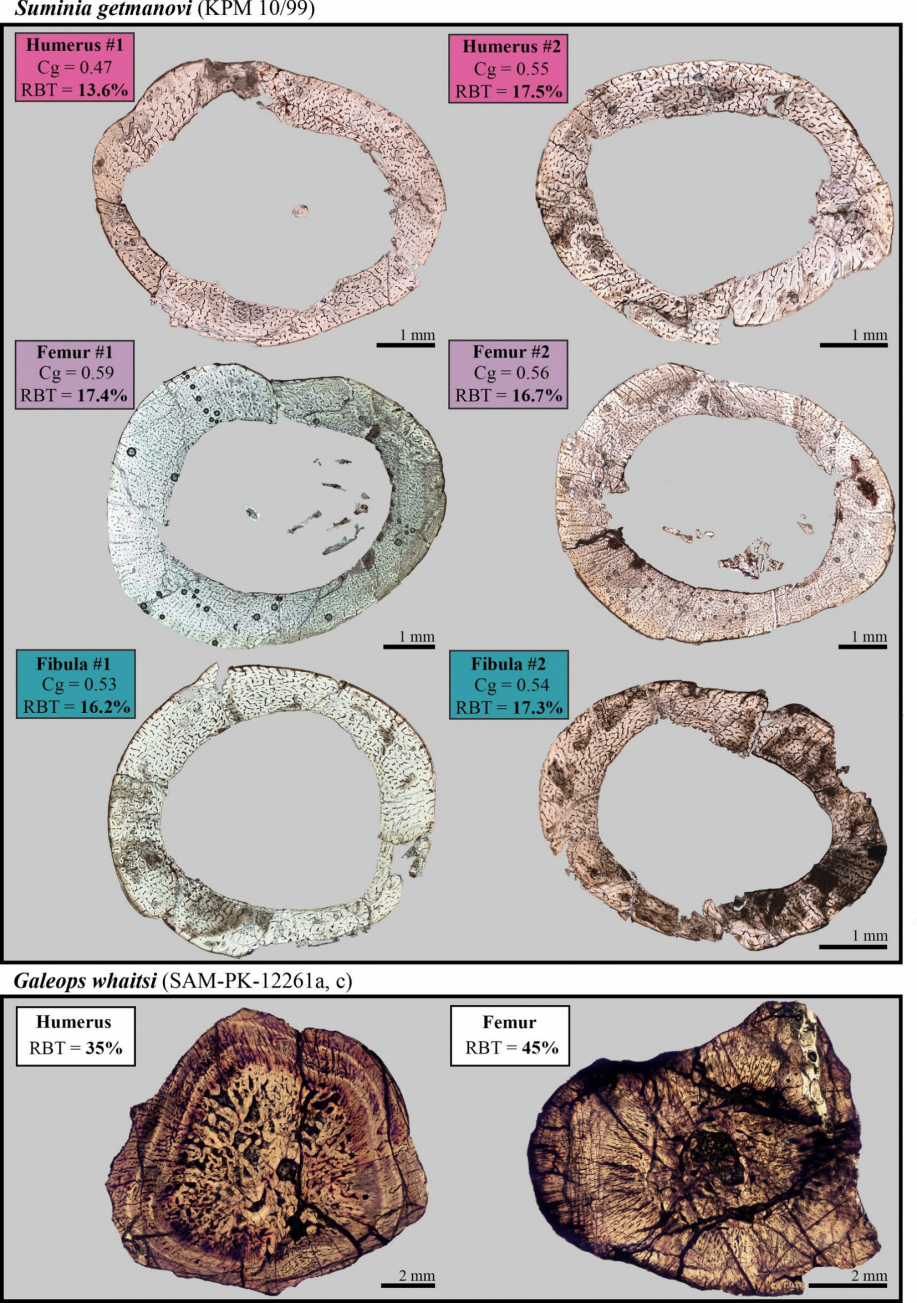
Comparative mid-diaphyseal limb bone microanatomy of two basal anomodonts, *Suminia getmanovi* (KPM 20/99; two retrodeformed sections (#1, #2) were obtained at the midshaft for the left humerus, the left femur and the fibula) and *Galeops whaitsi* (SAM-PK-12261a, c; thin-section images provided by J. Botha and B. Mark Weiss; RBT values from^18^. Cg, global compactness; RBT, relative bone wall thickness.

Regardless of the extent of diagenetic alteration, all sampled limb bones show a relatively thin, but compact cortex at the mid-diaphysis. The transition between the cortex and the medullary cavity is abrupt and thus well defined. The medullary cavity is large and virtually free of bone trabeculae (Fig. 6). This translates, for the retrodeformed sections, into a *Cg* up to 0.55 for the left humerus (section #2), 0.59 for the large left femur (#1), and 0.54 for the fibula (Fig. 6; Table 1). Each section also shows relatively low *S* values (≤ 0.034) and high *P* values (≥ 0.65), which reflect a narrow transition zone between the compact cortex and the medullary cavity and a relatively wide medullary cavity, respectively. Finally, the calculated RBT is less than 18% in all retro-deformed elements (left humerus, large left femur, fibula; Fig. 6; Table 1). This is also true for the CDI/2 values obtained in Bone Profiler that remain inferior to 18% (see Table 1).

**Table 1:** Linear measurements and microanatomical parameters of the studied skeletal elements.

Skeletal element	Humerus (L)		Femur (L)		Femur (R)	Tibia (R)	Fibula	
Section number	#1	#2	#1	#2	#1	#1	#1	#2
Lg [mm]	53.49		75.83		64.32	59.40	51.63	
Circ [mm]	18.76	16.44	20.09	22.15	–	–	–	–
Dm [mm]	5.46	4.64	6.03	6.27	–	–	4.76	4.46
Tm [mm]	0.74	0.81	1.05	1.04	–	–	0.77	0.77
RBT [%]	13.55	17.46	17.41	16.59	–	–	16.18	17.26
CDI	0.28	0.33	0.35	0.34	–	–	0.32	0.33
CDI /2 [%]	13.89	16.55	17.68	17.02	–	–	15.97	16.58
Cg	0.47	0.55	0.59	0.56	–	–	0.53	0.54
S	0.03	0.03	0.02	0.03	–	–	0.03	0.03
P	0.72	0.67	0.65	0.66	–	-	0.68	0.67

.

CDI, cortico-diaphyseal index; Circ, circumference; Cg, global compactness; Dm, diameter of the section; Lg, maximal length; P, parameter P reflecting the size of the medullary cavity; RBT, relative bone wall thickness; S, parameter S reflecting the size of the transition zone between the compact cortex and the medullary cavity; Tm, cortical thickness. Note that RBT values have been calculated as shown in Fig. S2F, following the method implemented in^18^. CDI values were directly obtained with Bone Profiler^30^.

### Outcome of inference models

*Suminia* was reconstructed as terrestrial by the models of Canoville and Laurin^41^ and Quemeneur et al.^42^ based on humeral (section #2) and femoral (large femur, section #1) microanatomical parameters, respectively (Table 2). The model of Gônet et al.^43^ inferred a sprawling posture of the forelimb of *Suminia* based on its humeral (section #2) overall structure (Table 2). Based on both equations established by Campione and Evans^44^ to estimate quadrupedal terrestrial tetrapod body mass, a weight of 1.6 kg was calculated for the individuals represented by specimen KPM 20/99 (see Table 2).

**Table 2:** Inference models used and the corresponding predictive parameter values for the selected cross-sections.

Model used	^41^	^42^	^43^	^44^
Inference type	Lifestyle (terrestrial, amphibious, aquatic)	Lifestyle (terrestrial, amphibious, aquatic)	Posture (sprawling, semi-erect, erect)	Body mass
Bone considered	Humerus (#2)	Femur (#1)	Humerus (#2)	Humerus (#2) & Femur (#1)
Parameters	S = 0.029 P = 0.669 Min = 0.000 Max_rad_ = 1.000 Cc = 0.000 Cp = 1.000	S = 0.019 Max_rad_ = 1.000 Min_rad_ = 0.028 C_m_ = 0.59 Min = 0.035 Cr = 0.59 SVL = 30–35 cm	Circ/Pe_min_ = 16.44 [mm] P = 0.669 SR = 37.05	Circ humerus = 16.44 mm Circ femur = 20.09 mm
Inference Results	Terrestrial	Terrestrial	Sprawling	1.6 kg

## Discussion

The size and microstructure of the considered limb bones indicate that KPM 20/99 comprises skeletal elements of at least two individuals. Indeed, the limb bone length ratios suggest that the larger left femur and the right tibia may have belonged to a single individual (or two similar-sized animals), more than 93% the size of one of the largest known individuals of *Suminia getmanovi* (PIN 2212/116, Specimen 5) hypothesized as adult by Fröbisch and Reisz^24^ based on morphology, body-size, and the co-ossification of some elements in the manus. The smaller right femur and the fibula are both about 80% of the corresponding elements of PIN 2212/116, Specimen 5. These bones fall within the size range of the smallest individuals of PIN 2212/116 that have been interpreted as subadults based on morphology^24^. The limb proportion data are not conclusive for the sampled humerus.

The osteohistological results confirm that at least the femora belonged to individuals that died at slightly different ages. Indeed, the larger left femur exhibits osteohistological features associated with a more advanced development than the smaller right femur. These include a marked decrease in vascularization and osteocyte lacuna density in the outer quarter of the cortex, coupled with a LAG close to the periosteal surface, testifying that growth had already slowed down and that the animal’s initial uninterrupted growth burst was over. The smaller right femur only shows a very slight decrease in growth rate in the outermost cortex, without any visible LAG. Secondary remodeling, as seen by the presence of secondary osteons, was more pronounced in the larger left femur.

The bone microstructure further indicates that all sampled bones belonged to individuals that died at a late juvenile to early subadult stage. All elements showed at least a slight decrease in growth rate in the outermost cortex, with the deposition of a region of bone containing better organized, more sparse and flattened osteocyte lacunae than the subjacent bone tissue. These bones were thus past the initial burst of growth encountered in very young juveniles. However, none of these elements belonged to a somatically mature (i.e. fully grown) individual. Vascular canals reach the bone surface in all skeletal elements. All the bones, including the larger femur, exhibit resorptive endosteal margins and no deposition of endosteal circumferential lamellae, which may indicate an older ontogenetic stage in continental tetrapods^29^, including anomodonts^18^. However, it may also indicate that the peri-medullary region was being resorbed at the time of death as the inner bone surface is constantly remodeled during life and thus, the presence of endosteal circumferential lamellae may not be a good indicator of age.

Our interpretations regarding the ontogenetic stage of the sampled individuals do not fully support the hypothesis of Fröbisch and Reisz^24^. Our sample fits within the size range documented among the 15 individuals preserved together in PIN 2212/116 and interpreted as subadult to fully grown adults by these authors^24^. However, histological examinations reveal that the bones sampled in our study belonged to late juveniles to early subadults, which in turn suggests that the 15 individuals of PIN 2212/116 might have been slightly younger than hypothesized by Fröbisch and Reisz^24^.

Our osteohistological observations correspond to what has been described in other anomodonts^18^. Botha and Angielczyk^18^ showed that in the vast majority of the species, initial growth is fast and continuous up to the subadult stage and that LAGs only appeared late in ontogeny when the animals were already 50–70% of adult size. The limb bones in our sample all exhibit relatively well-vascularized cortices (with some noticeable radial canals or radial anastomoses related to fast accretion rates;^29^), made of woven-parallel complex and/or parallel-fibered bone tissue, indicative of a relatively high and uninterrupted growth rate up to about 80% (as shown in the small right femur and the fibula) of the size of the largest documented *Suminia* specimens^24^. These results suggest that *Suminia getmanovi* had a growth strategy similar to other anomodonts, and especially more derived dicynodonts.

Based on our sample, we calculated a body mass of 1.6 kg for late juvenile to early subadult individuals of *Suminia getmanovi*. We recognize that this estimate is an approximation since our calculations rely on the humeral and femoral circumferences of individuals that may have died at slightly different stages in their development and were not fully grown (> 80% of the largest known individual size). However, this body mass estimate confirms that *Suminia* was among the most light-weighted anomodonts that ranged in body mass from below one kilogram, such as *Patranomodon* or *Pristerodon*^11^, up to several hundred kilograms like *Endothiodon*^45^ or even several tons like *Lisowicia*^17,46^. The small size of *Suminia* is advantageous for an arboreal lifestyle, as less energy is required to lift the body weight against gravity, as observed in extant scansorial reptiles and mammals, such as primates and carnivorans that tend to be smaller than their terrestrial counterparts^47^.

Based on its humeral proportions and microanatomy, *Suminia* was retrieved as having a sprawling forelimb with the model in^43^. Such result was expected since morphological and biomechanical studies^48,49^ have already demonstrated that anomodonts had a sprawling forelimb and that the transition to a parasagittal posture occurred much later in the evolutionary history of Synapsida^10^. Previously published lifestyle inference models based on humeral^41^ and femoral^42^ microanatomy were applied to our *Suminia* sample. Unfortunately, available models based on large comparative microanatomical datasets were not built to discriminate between arboreal taxa and their terrestrial or aquatic relatives. Unsurprisingly, *Suminia* was thus recovered as a terrestrial animal based on its microanatomy, which is closest to the tubular limb bone structure seen in extant non-graviportal terrestrial tetrapods and strongly departs from the more robust limb bone structure of amphibious and aquatic taxa inhabiting shallow waters^31^.

Indeed, all sampled stylopodial and zeugopodial elements present a consistent bone microanatomy at the midshaft: i.e., a compact, but relatively thin cortex and a wide-open medullary cavity (Fig. 6). Besides, the transition zone between the deep cortex and the medullary cavity is well-defined and abrupt (resulting in low *S* values) and overall RBT values are below 18%. The microanatomy of late juveniles to early subadult individuals of *Suminia* studied here is thus in contrast with the bone structure of its closest relatives at similar ontogenetic stages. *Galeops whaitsi*, the only other non-dicynodont anomodont previously investigated histologically (see Fig. 6) and all other dicynodonts, considered terrestrial and/or fossorial, exhibit, independently of size and ecology, much more compact limb bones^15,18^. In these species, RBT values systematically exceed 30% in subadult and adult individuals^18^. Additionally the transition from the medullary cavity to the compact cortex in anomodonts is often progressive and the medullary cavity usually filled with trabeculae.

Synapsid groups more basal than anomodonts tend to also exhibit a medullary area invaded by trabeculae and/or higher cortical thickness values than *Suminia* in their limb bones. For instance, dinocephalians, whether inferred terrestrial or semi-aquatic, are all characterized by thick bone walls and a medullary area completely infilled with trabeculae, resulting in relatively high global compactness values^50^. Pelycosaur-grade synapsids also tend to show high compactness values and/or a medullary area obstructed by trabecular bone (e.g.^51^). Although the presence of trabeculae in the medullary cavity might be partly explained by the large body size of some of these species^34,52^, smaller taxa, such as *Dimetrodon natalis*^51^, also show a well-developed trabecular network in the shaft of their limb bones. Even varanopids that were agile terrestrial predators with slender limbs and tubular long bones with open medullary cavities^53,54^ and low *S* values (^53^: Table 1), tend to show slightly higher RBT values [> 20%; see^53^: Table 1 where RBT = (CDI/2)*100] than *Suminia.* It is interesting to note, nonetheless, that at least two varanopid species, *Ascendonanus* from the early Permian of Germany^55^ and *Eoscansor cobrensis* from the late Carboniferous of New Mexico, USA^56^, have now been inferred as potentially even older arboreal tetrapods based on morphology and body proportions convergent with *Suminia* and might therefore also exhibit similar limb bone structure. However, their long bone microanatomy remains to be investigated.

The long bone microanatomy of *Suminia* translates into an overall lightening of the skeleton as compared to other related species. It is thus completely unique among anomodonts, and also departs from the expected plesiomorphic condition of the clade. 

Arboreal vertebrates are a major component of terrestrial ecosystems. Several lineages of amphibians, reptiles, and synapsids (including mammals) have repeatedly and independently adapted to life in trees and evolved convergent morphological specializations for grasping, clinging, and hooking that have been extensively studied (e.g.^57,58^). However, only a few researchers have examined the effect of an arboreal lifestyle on the limb bone mid-diaphyseal structure of these animals. Notable studies^36,37^ showed that, as seen in *Suminia*, scansorial mustelids possess lightened long bones with thinner bone walls and lower compactness values than their terrestrial and aquatic relatives.

Together with a long list of postcranial adaptations (e.g., a long tail, slender limbs and elongated phalanges, long and laterally compressed unguals, divergent first digit in manus and pes;^21,24^) and a highly specialized feeding apparatus to process high-fiber plant material such as leaves^27^, bone microstructure comes as an additional line of evidence for an arboreal lifestyle in this very unique anomodont therapsid.

## Material and methods

### Fossil material and its provenance

Our sample consists of five isolated long bones recovered by the team of the Vyatka Paleontological Museum from the Kotel’nich locality of the Kirov region of Russia, whose geological age is debated and has been alternatively assigned to the early Late Permian (Wuchiapingian;^21^) or the late Middle Permian (Capitanian;^25^). Its red beds have been a source of exceptionally well-preserved fossil tetrapods (see^24^: table S2;^25^).

The skeletal elements of interest were all assigned to the specimen number KPM 20/99 and were given to A. Canoville for destructive sampling in 2012 by the Kirov Paleontological Museum, Kirov, Russia. All necessary approvals were obtained to study this material. All bones are heavily crushed dorso-ventrally, but can be clearly referred to the small anomodont *Suminia getmanovi* (see^21^ for details on *S. getmanovi* postcranial anatomy) and consist of (1) a left humerus [53.49 mm in total length (***Lg***); Fig. 2B]; (2) a left femur (*Lg* = 75.83 mm; Fig. 2C); (3) a right femur (*Lg* = 64.32 mm; Fig. 2D); (4) a right tibia (*Lg* = 59.4 mm; Fig. 2E); (5) and a fibula (*Lg* = 51.63 mm; Fig. 2F) (Table 1).

### Size range and limb bone proportions

In order to test whether these five skeletal elements belonged to a single individual, we compared the limb bone proportions of our sample to those of several articulated and nearly complete specimens of *Suminia getmanovi*, including (1) the 15 articulated to semi-articulated individuals of PIN 2212/116 studied in [^21^: appendix 1]; (2) the forelimb of PIN 2212/62 figured in [^21^: fig. 9]; (3) the left hindlimb of PIN 2212/102 figured in [^21^: fig. 13]; (4) the right forelimb of ROM 80979 measured by A. Canoville at the Royal Ontario Museum, Toronto, Canada. All linear measurements are recorded in Table S1 and the ratios between skeletal elements are presented in Table S2. We also compared the length of the studied skeletal elements to their documented size range in *S. getmanovi* (Fig. S1A), based on the comparative material mentioned above and the measurements provided in [^21^: appendix 1].

### Record of 3D gross morphology before destructive sampling

To preserve a record of their 3D gross morphology and microanatomy, each skeletal element was first micro CT-scanned individually at the Museum für Naturkunde, Berlin, using a FF85 dual-tube system (YXLON International, Hamburg, Comet Group). The long bones were scanned in their entirety with a 190 kV multifocus tube in microfocus mode. Higher-magnification scans of the mid-shafts were also made using the nanofocus mode. All virtual sections were then obtained and analyzed in VG Studio Max 3.4 (Volume Graphics GmbH, Heidelberg, Germany). The different skeletal elements were also photographed and carefully measured using a digital caliper (Table 1). The mid-diaphyseal region to be sampled was then molded using a dental silicone (Vinylpolysiloxane precision impression material) from Provil novo (Putty soft regular set) that cured in about three minutes and could then be carefully removed from the specimen.

### Destructive sampling and subsequent bone repair

A roughly 0.5 cm thick slice of bone was removed from the mid-diaphysis of the different skeletal elements using a NSK Ultimate XL Micromotor equipped with JOKE diamond tools and specialty diamond blades (9-S1031 by Botzian & Kirch). Sampling was restricted to the location of the growth center (which is around mid-diaphysis in these skeletal elements, as observed on the CT-scans) because it corresponds to the portion of the shaft where the cortex is the thickest and growth record is maximal^29^. Several studies have also shown that bone microanatomy usually varies along the shaft of long limb bones^29,60^ and that, at the level of the growth center, inner bone architecture contains the highest ecological signal^61^. The sampled portions were reconstructed using a kneadable epoxy (Aves APOXIE Sculpt Modeling compound) using the silicone molds that were made before destructive sampling.

### Thin-sectioning protocol and image acquisition

We processed ground-sections following standard petrographic protocols^62^ at the Friedenstein Stiftung Gotha, Gotha, Germany. The extracted bone samples were embedded in EPO-TEK® 301-1 resin (Epoxy Technology Inc). For each skeletal element, two to five wafers of embedded bone were then cut using a Buehler IsoMet Low Speed Saw equipped with a Buehler IsoMet Wafering Blade (Series 15HC; No. 11-4245), affixed to frosted petrographic glass slides with epoxy, and finally ground to desired thickness (100–80 μm) with a Buehler EcoMet 30 polisher and Buehler CarbiMet SiC abrasive papers with decreasing grit sizes (P240, P400, P800, P1200). Ground sections were examined and photographed using a Zeiss Axioscope 7 microscope equipped with an Axicocam 305 color camera. Histological images were captured under normal and cross-polarized (with or without a λ-compensator) light using the software ZEN Core v3.3.

### Retrodeformation of cross-sections and quantification of bone microanatomy

All studied skeletal elements were crushed during fossilization, as it is typical for the long bones of *Suminia* (Fig. S2A; J. Fröbisch pers. obs.). In order to extract quantitative parameters describing the long bone cross-sectional shape and inner architecture, we had to first retro-deform, when possible, the cross-sections using Adobe Photoshop 2020 (Fig. S2B). In short, we manually isolated the different cortical pieces as individual layers, searched for the best connections between them, and finally stitched them together to get a fully reconstructed section. In order to test for the reproducibility of the method, two of us carried out these steps individually, and obtained similar reconstructions. The smaller right femur and the right tibia were too diagenetically altered and fragmentary to reconstruct their original cross-sectional shape. However, two sub-complete and retro-deformed sections (#1 and #2) were obtained for the left humerus, the left femur, and the fibula, respectively (Table 1; Fig. 6). All retro-deformed sections were then converted into binary images (black for bone and white for voids, i.e. resorption cavities, medullary cavity; Fig. S2C).

From these retro-deformed sections, we extracted different parameters that are recorded in Table 1: (1) the mean diameter (***Dm***) of the section that corresponds to the average of four diameters measured as shown on Fig. S2E; (2) the mean cortical thickness (***Tm***) of the section that corresponds to the average of eight bone wall thickness measurements as shown on Fig. S2E; (3) the relative bone wall thickness (***RBT***) previously used in the literature, especially for anomodonts^18^. The *RBT* is expressed as a percentage and calculated as the ratio between *Tm* and *Dm* (Fig. S2F). A low *RBT* value corresponds to a relatively thin cortex, whereas higher *RBT* values indicate thicker cortices; (4) the cortico-diaphyseal index (***CDI***), which corresponds to the thickness of the cortex divided by the radius of the bone, was also calculated in Bone Profiler^30^ along 60 different transects of the section. When the cortical thickness varies within the section, *CDI* calculations obtained in Bone Profiler are thus more accurate than *RBT* calculations. For a given section, *RBT* should approximate *CDI*/2; (5) the outer circumference of the cross-section (***Circ***) calculated in ImageJ; some compactness profile parameters obtained with Bone Profiler (for details see^30^; see also Fig. S2D) and describing the bone tissue organization within the cross-sections, such as (6) ***Cg*** corresponding to the global compactness of the bone cross-section; (7) the extension of the medullary cavity (parameter ***P***); (8) the width of the transition zone between the compact cortex and the medullary cavity (parameter ***S***). *S* tends to be high in the presence of trabeculae between the free medullary cavity and the compact cortex and low when trabeculae are absent. For a few sections used in the inference models (see below; Table 2), we also obtained in Bone Profiler: (9) ***Min*** and ***Min***_***rad***_, both corresponding to the value of the lower asymptote of the curve and computed from a single global compactness profile or the average of 60 profiles, respectively. These parameters reflect the compactness in the center of the section and equal zero when the medullary cavity is completely free of bone trabeculae; (10) ***Max*** and ***Max***_***rad***_, both corresponding to the value of the upper asymptote of the curve and computed from a single global profile or the average of 60 profiles, respectively. These parameters reflect the compactness in the outer cortex; (11) ***Cc***, representing the compactness in the center of the bone section; (12) ***Cp*** reflecting the compactness at the periphery of the cross-section. Finally, the slenderness ratio (***SR***), which describes the overall proportions of the long bone from slender (high *SR*) to more robust (low *SR*), was also calculated (as defined in^43^) for the left humerus (see Table 2). Note that all values obtained for these parameters are approximations, considering that the long bones were crushed and fragmentary and that small cortical fragments might be missing on the retro-deformed sections.

### Inference models

Several inference models available in the literature were applied to the long bones studied here (see Table 2). We used the lifestyle inference models from^41^ and^42^ to predict the lifestyle of *Suminia getmanovi* based on its humeral and femoral microanatomy, respectively. The Canoville and Laurin^41^ model is built off microanatomical data extracted from the adult humeri of 75 extant and 9 extinct amniote taxa of known lifestyle (terrestrial, amphibious, aquatic). The authors found that lifestyle can be best predicted from 6 compactness profile parameters, i.e. *S*, *P*, *Min*, *Max*_*rad*_, *Cc*, *Cp* (Table 2). We chose section #2 (closer to the growth center with a smaller diameter and a higher cortical thickness than section #1) of the humerus to apply this model. Quemeneur et al.^42^ built their model from body size measurements and femoral microanatomical parameters gathered from 155 extant amniotes of known lifestyle (terrestrial, amphibious, aquatic). These authors found that a combination of 7 parameters best predicted lifestyle, i.e. the species snout-vent-length (*SVL*), *S*, *Max*_*rad*_, *Min*, *Min*_*rad*_, *Cg*, modeled compactness *Cm* (Table 2). We chose section #1 (closer to the growth center with a smaller diameter and a higher cortical thickness than section #2) of the largest femur (left) to apply this model. Unfortunately, there is currently no model available in the literature to discriminate arboreal tetrapods based on microanatomy. While these two models do not consider an arboreal lifestyle, they can be used to show that *Suminia* has a microanatomy closer to extant terrestrial amniotes than amphibious and aquatic ones. In order to use the model in^41^, we had to assess the snout-vent-length of *Suminia getmanovi.* Fröbisch and Reisz^21^ estimated that most individuals preserved on the slab specimen PIN 2212/116 were about 50 cm in total body length. Also, as described in^21,24^, *Suminia* had an unusually long tail made of 52 caudals (^21^: table 1) and representing about 30–40% of the total body length (see specimen 2 of PIN 2212/116 in fig. 1, as well as the skeletal reconstruction of *Suminia* in fig. 16 of^21^). We thus estimated *Suminia*’s SVL to be approximately 30 to 35 cm.

We also used the model in^43^ to infer the posture (sprawling, semi-erect, erect) of *Suminia getmanovi*. This model is based on humeral cross-sections gathered from 41 extant synapsid species. The authors found three humeral parameters to be good predictors of the humeral posture, i.e. the minimum perimeter of the shaft (*Pe*_*min*_), which corresponds to the circumference (*Circ*) of the humerus we measured at midshaft, parameter *P*, and *SR*. We applied this model to section #2 of the humerus (Table 2).

Finally, we used the equations established by Campione and Evans^44^ to estimate *Suminia*’s body mass based on stylopodial circumferences (Table 2). Their equations are robust predictors of body mass in quadrupedal terrestrial tetrapods despite their diverse postures, gaits, and phylogenetic affinities.

## Supplementary Information

Supplementary Information 1.

Supplementary Information 2.

Supplementary Information 3.

Supplementary Information 4.

## Data Availability

The complete datasets generated and/or analyzed during the current study are available from the corresponding author on reasonable request. High-resolution renders of the different mid-diaphyseal cross-sections done for this study are also available on request through MorphoSource (project ID 000699917) at https://www.morphosource.org/projects/000699917.
