# Supplementary material for: Bone microstructure of the basal anomodont Suminia getmanovi supports its arboreal lifestyle
Source: Sci Rep. 2025 Mar 25;15:10294. doi: 10.1038/s41598-025-92727-w (PMC11937274; doi:10.1038/s41598-025-92727-w)
Supplement: Supplementary file 1 — Supplementary Information 1. [file 41598_2025_92727_MOESM1_ESM.pdf]

Figure S1. Limb bone length and proportions in *Suminia getmanovi*. **A**: Size range known to date for the humerus (a), femur (b), tibia (c), and fibula (d) in *Suminia getmanovi.* KPM skeletal elements under study are highlighted in blue. The size of these bones is given as a percentage of the size of the largest specimen known for this taxon. **B**: Box plots of the limb bone length ratios calculated from articulated individuals (specimens PIN 2212/116, PIN 2212/62, PIN 2212/102, ROM 80979) and ratios calculated for specimen KPM 10/99. Abbreviations: F, femoral length; Fi, fibular length; H, humeral length; T, tibial length.

A.

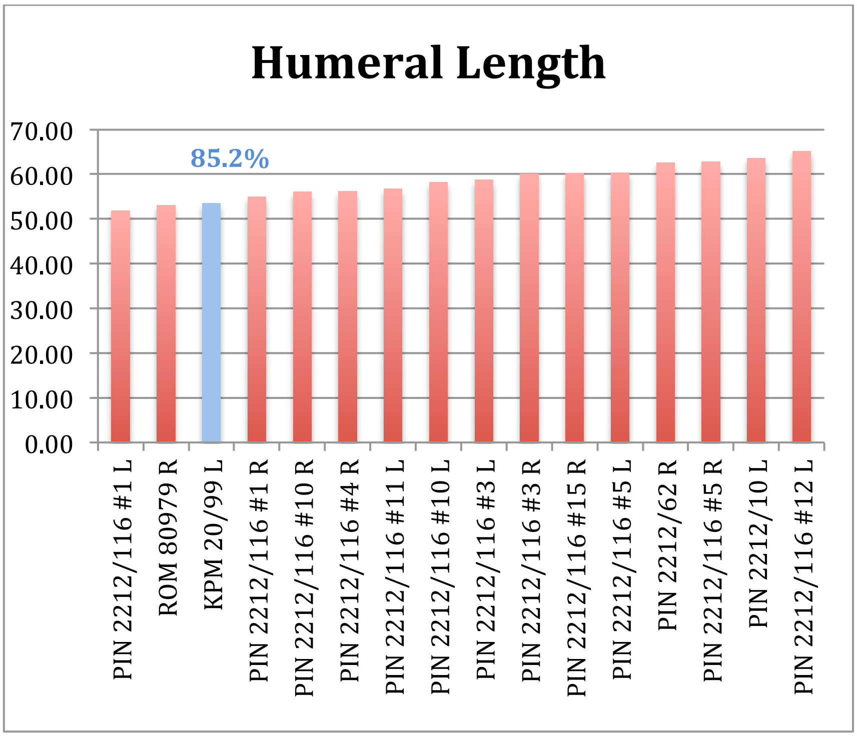

a)

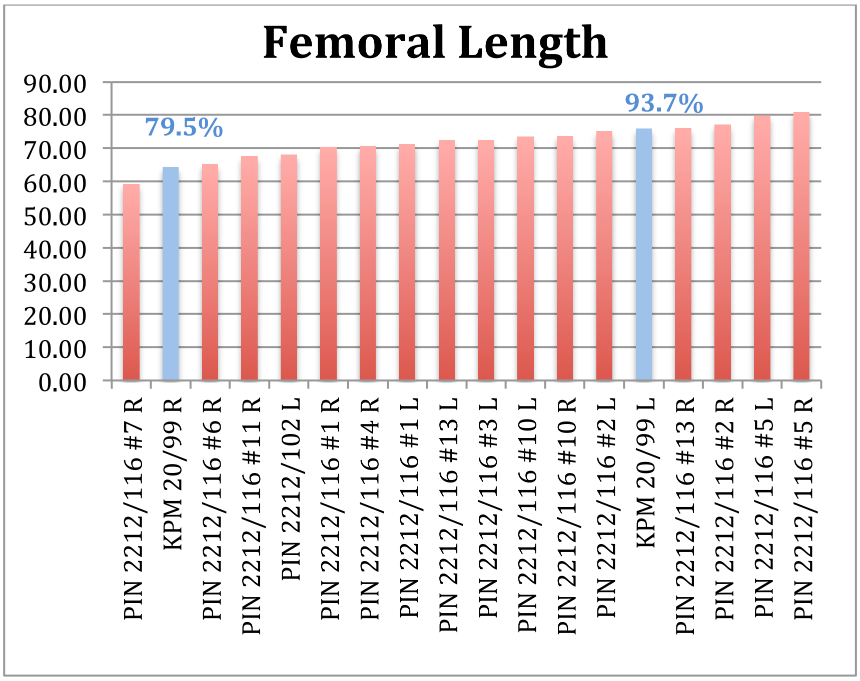

b)

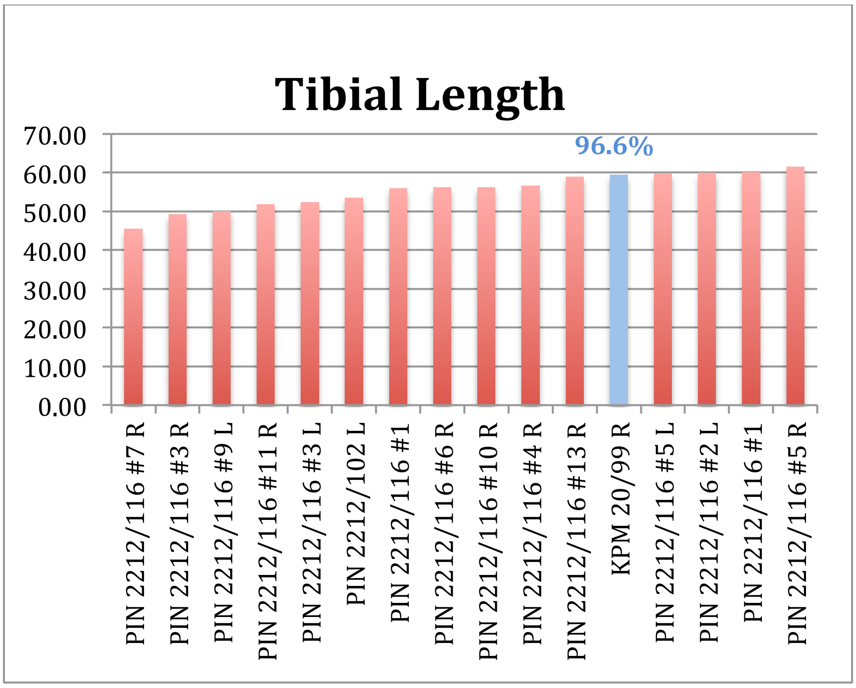
Tibial Length
70.00
60.00
50.00
40.00
30.00
20.00
10.00
0.00
96.6%
PIN 2212/116 #7 R
PIN 2212/116 #3 R
PIN 2212/116 #9 L
PIN 2212/116 #11 R
PIN 2212/116 #3 L
PIN 2212/102 L
PIN 2212/116 #1
PIN 2212/116 #6 R
PIN 2212/116 #10 R
PIN 2212/116 #4 R
PIN 2212/116 #13 R
KPM 20/99 R
PIN 2212/116 #5 L
PIN 2212/116 #2 L
PIN 2212/116 #1
PIN 2212/116 #5 R

c)

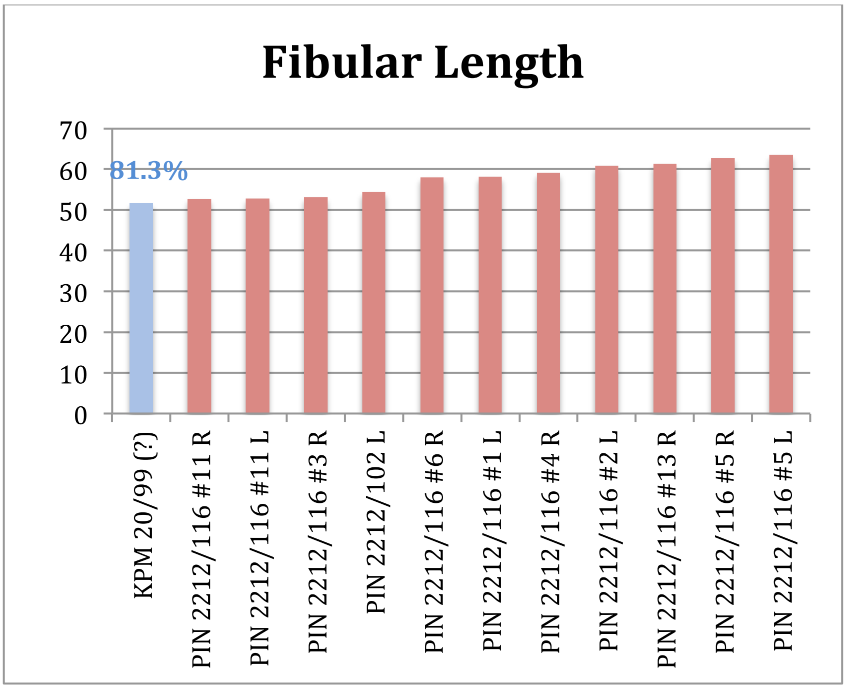
Fibular Length
70
60
50
40
30
20
10
0
81.3%
KPM 20/99 (?)
PIN 2212/116 #11 R
PIN 2212/116 #11 L
PIN 2212/116 #3 R
PIN 2212/102 L
PIN 2212/116 #6 R
PIN 2212/116 #1 L
PIN 2212/116 #4 R
PIN 2212/116 #2 L
PIN 2212/116 #13 R
PIN 2212/116 #5 R
PIN 2212/116 #5 L

d)

B.

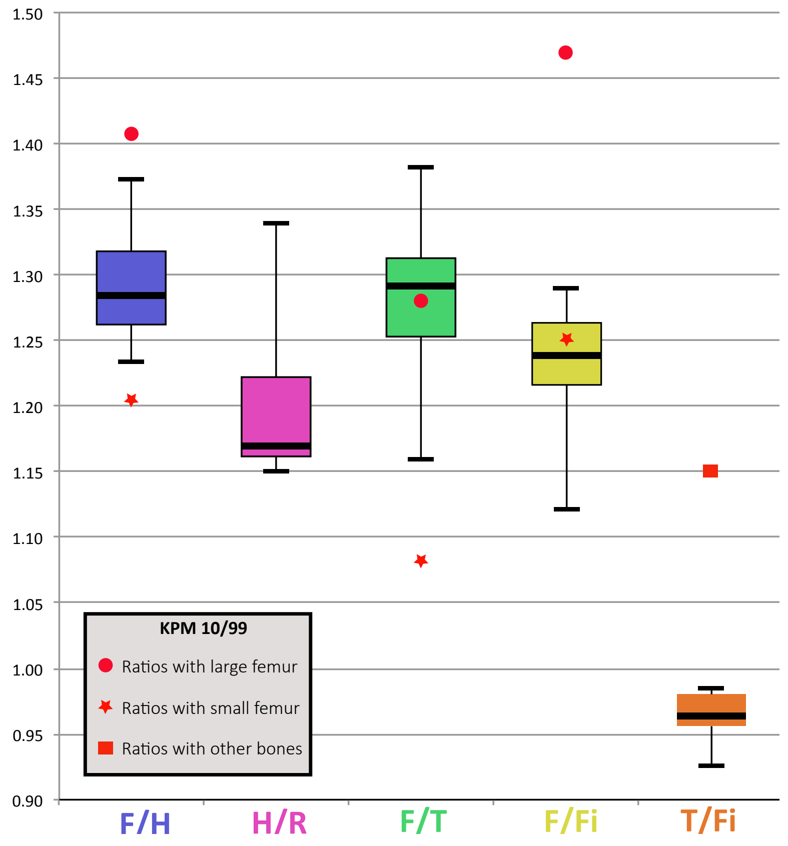

1.50
1.45
1.40
1.35
1.30
1.25
1.20
1.15
1.10
1.05
1.00
0.95
0.90
KPM 10/99
Ratios with large femur
Ratios with small femur
Ratios with other bones
F/H
H/R
F/T
F/Fi
T/Fi
