# Supplementary material for: Bone microstructure of the basal anomodont Suminia getmanovi supports its arboreal lifestyle
Source: Sci Rep. 2025 Mar 25;15:10294. doi: 10.1038/s41598-025-92727-w (PMC11937274; doi:10.1038/s41598-025-92727-w)

Figure S2. Microanatomical analysis of the long bone diaphyseal cross-sections of specimen KPM 10/99. The diagenetically-altered cross-sections (**A**) were, when possible, retrodeformed in Adobe Photoshop 2020 (**B**) and then converted into binary images (black for bone and white for voids; **C**). Such images were analyzed in Bone Profiler to obtain a compactness profile describing the bone tissue organization within the cross-sections (**D**). The mean diameter (*Dm*) and mean cortical thickness (*Tm*) of the section that correspond, respectively, to the average of four diameters and height cortical thickness measurements as shown in (**E**) were used to calculate the relative bone wall thickness (RBT) following the formula in (**F**).

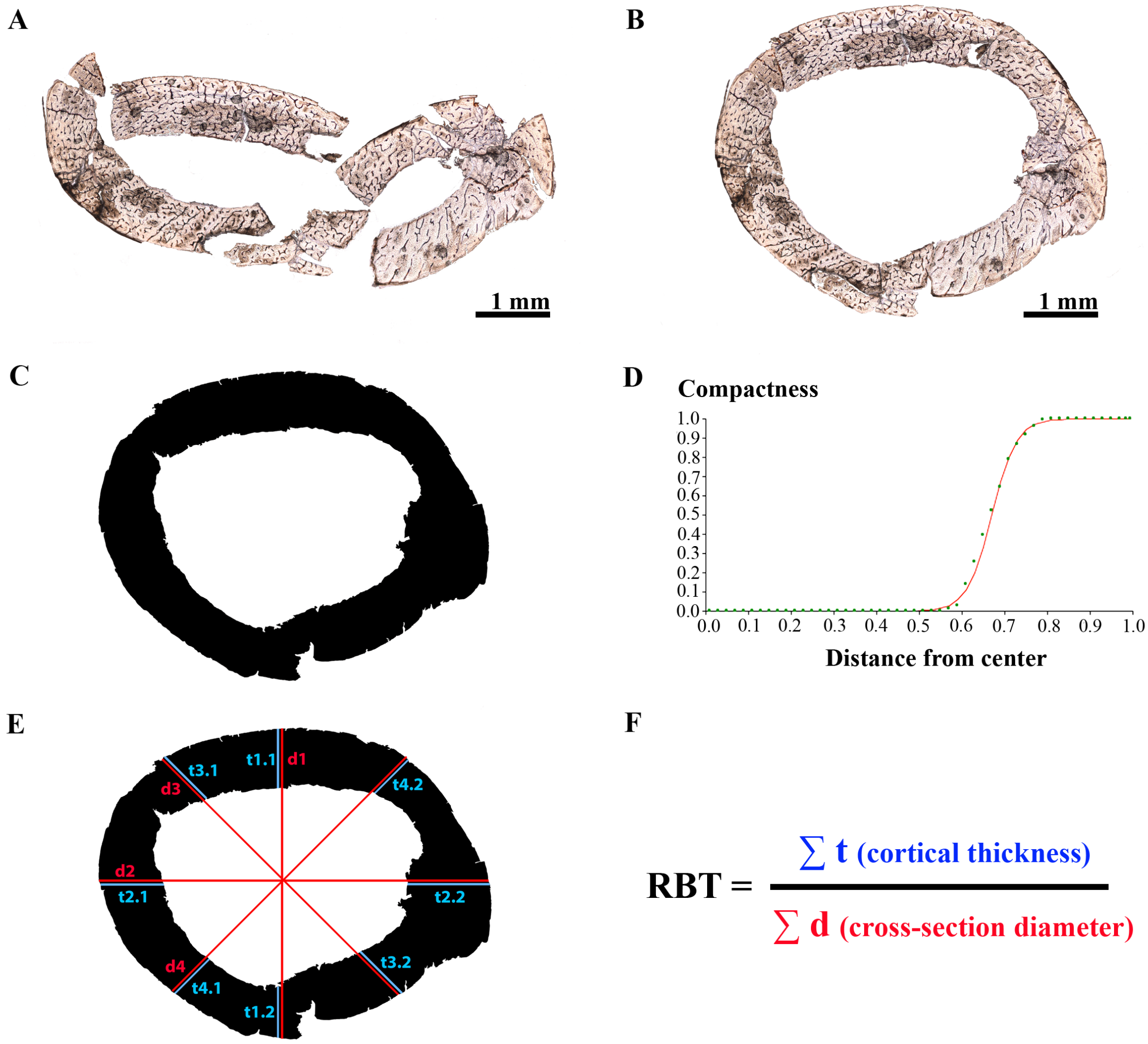

Supplement: Supplementary file 2 — Supplementary Information 2. [file 41598_2025_92727_MOESM2_ESM.pdf]
